# AxPcoords & parallel AxParafit: statistical co-phylogenetic analyses on thousands of taxa

**DOI:** 10.1186/1471-2105-8-405

**Published:** 2007-10-22

**Authors:** Alexandros Stamatakis, Alexander F Auch, Jan Meier-Kolthoff, Markus Göker

**Affiliations:** 1 École Polytechnique Fédérale de Lausanne, School of Computer & Communication Sciences, Laboratory for Computational Biology and Bioinformatics STATION 14, CH-1015 Lausanne, Switzerland; 2 Swiss Institute of Bioinformatics; 3 Center for Bioinformatics (ZBIT), Sand 14, Tübingen, University of Tübingen, Germany; 4 Organismic Botany/Mycology, Auf der Morgenstelle 1, Tübingen, University of Tübingen, Germany

## Abstract

**Background:**

Current tools for Co-phylogenetic analyses are not able to cope with the continuous accumulation of phylogenetic data. The sophisticated statistical test for host-parasite co-phylogenetic analyses implemented in Parafit does not allow it to handle large datasets in reasonable times. The Parafit and DistPCoA programs are the by far most compute-intensive components of the Parafit analysis pipeline. We present AxParafit and AxPcoords (Ax stands for Accelerated) which are highly optimized versions of Parafit and DistPCoA respectively.

**Results:**

Both programs have been entirely re-written in C. Via optimization of the algorithm and the C code as well as integration of highly tuned BLAS and LAPACK methods AxParafit runs 5–61 times faster than Parafit with a lower memory footprint (up to 35% reduction) while the performance benefit increases with growing dataset size. The MPI-based parallel implementation of AxParafit shows good scalability on up to 128 processors, even on medium-sized datasets. The parallel analysis with AxParafit on 128 CPUs for a medium-sized dataset with an 512 by 512 association matrix is more than 1,200/128 times faster per processor than the sequential Parafit run. AxPcoords is 8–26 times faster than DistPCoA and numerically stable on large datasets. We outline the substantial benefits of using parallel AxParafit by example of a large-scale empirical study on smut fungi and their host plants. To the best of our knowledge, this study represents the largest co-phylogenetic analysis to date.

**Conclusion:**

The highly efficient AxPcoords and AxParafit programs allow for large-scale co-phylogenetic analyses on several thousands of taxa for the first time. In addition, AxParafit and AxPcoords have been integrated into the easy-to-use CopyCat tool.

## Background

One of the basic questions in evolutionary analyses [1] is whether parasites (e.g., lice or Papillomaviruses) or mutualists have co-speciated with their respective hosts (e.g., mammals). The constant accumulation of DNA and AA sequence data coupled with recent advances in tree building software, such as TNT [2], MrBayes [3], GARLI [4] or RAxML [5], allow for large-scale phylogenetic analyses with several hundred or thousand taxa [6-12]. Thus, large-scale co-phylogenetic studies have also potentially become feasible. However, most common co-phylogenetic tools or methods such as BPA, TreeMap or TreeFitter (see review in [13]) are not able to handle datasets with a large number of taxa or have not been tested in this regard with respect to their statistical properties. Therefore, there is a performance and scalability gap between tools for phylogenetic analysis and meta-analysis. The capability to analyze large datasets is important to infer "deep co-phylogenetic" relationships which could otherwise not be assessed [14].

Parafit [15] implements statistical tests for both overall phylogenetic congruence as well as for the significance of individual associations. Extensive simulations have shown that the Parafit tests are statistically well-behaved and yield acceptable error rates. The method has been successfully applied in a number of biological studies [16-19]. In addition, the Type-II statistical error of Parafit decreases with the size of the dataset (see [15]), i.e., this approach scales well on large phylogenies of hosts and associates. Due to these desirable properties, recent work on CopyCat [14] focused on improving the usability of Parafit via a Graphical User Interface (GUI) and automation of the analysis pipeline which transforms phylogenetic trees to patristic (tree-based) distance matrices, converts distance matrices to matrices of eigenvectors using DistPCoA [20], invokes Parafit, and parses input, intermediate, as well as output files. However, co-phylogenetic analyses with CopyCat can not be conducted on large datasets due to the excessive run time requirements of Parafit and DistPCoA, which represent the by far most compute-intensive part of the CopyCat analysis pipeline.

Here we present AxParafit and AxPcoords which are highly optimized and parallelized versions of Parafit and DistPCoA respectively. As outlined by the case-study on smut fungi on page 6 these accelerated programs allow for more thorough large-scale co-phylogenetic analyses and extend the applicability of the approach by 1–2 orders of magnitude, thus closing the aforementioned performance gap concerning current phylogenetic meta-analysis tools. Coupled with the easy-to-use CopyCat tool AxParafit/AxPcoords facilitate statistical co-phylogenetic analyses on the largest trees that can currently be computed.

## Implementation

For programming convenience and portability as well as due to the structure of the original Fortran code we re-implemented Parafit and DistPCoA in C from scratch.

### Sequential Optimization

The sequential C code was optimized by reducing unnecessary memory allocations for matrices in AxPcoords/AxParafit and using a faster method to permute matrices in AxParafit.

Thereafter the compute-intensive for-loops in AxParafit/AxPcoords were manually tuned. After those initial optimizations we profiled both programs and found that the run-times were now largely dominated (over 90% of total execution time) by a dense matrix-matrix multiplication in AxParafit and the computation of eigenvectors/eigenvalues in AxPcoords respectively. To further accelerate the programs we integrated function calls to the highly optimized matrix multiplication of the BLAS (Basic Linear Algebra Package [21]) package and eigenvector/eigenvalue decomposition in LAPACK (Linear Algebra PACKage [22]).

For BLAS we assessed the usage of ATLAS BLAS (Automatically Tuned Linear Algebra Software, math-atlas.sourceforge.net) as well as the ACML BLAS (AMD Core Math Library [23]) libraries on a 2.4 GHz AMD Opteron CPU. The ACML package showed slightly faster speeds (≈ 7–9%). However, AxParafit also provides an interface to the INTEL MKL (Math Kernel Library) and ATLAS BLAS implementations. AMD ACML, INTEL MKL, and ATLAS are all freely available for academic use. AxParafit can also be compiled without BLAS and rely on a manually tuned matrix multiplication which is approximately 4 times slower.

AxPcoords can use either the LAPACK functions implemented in the AMD ACML or INTEL MKL libraries. In addition, AxPcoords can also make use of the GNU scientific library [24] for eigenvector/eigenvalue computations.

**Figure 1:**
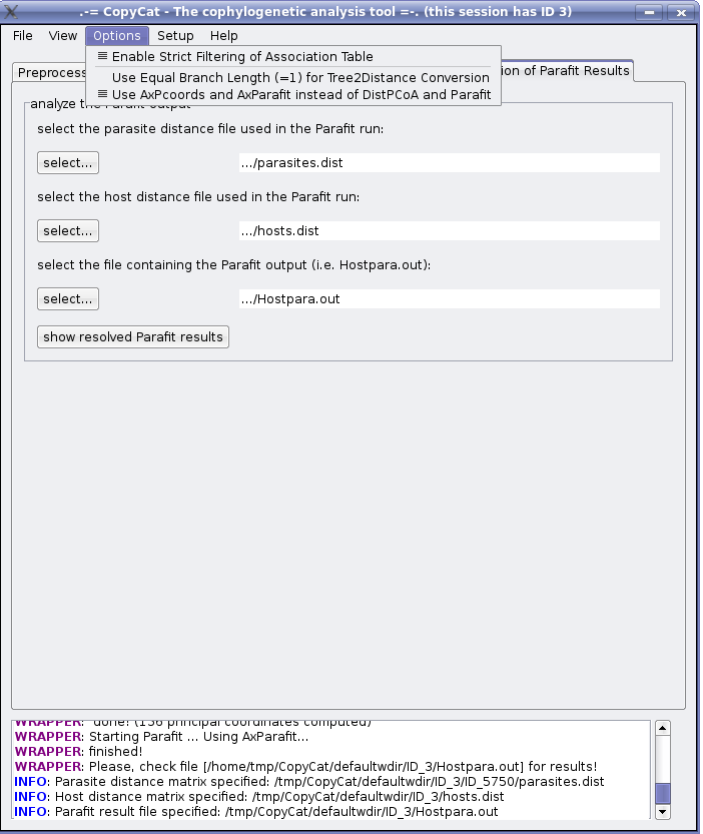
**Screen-shot of AxParafit/AxPcoords Option in CopyCat**. This screen-shot shows the CopyCat drop-down menu that allows the user to select AxParafit/AxPcoords for executing the analyses and to switch between the U and W modes of branch length computation.

The tuned programs were designed to yield *exactly *the same results as Parafit and DistPCoA. Note however, that in contrast to AxPcoords we observed numerically unstable results for DistPCoA on datasets with large association matrices, containing more than 4,096 entries. This is due to some well-known problems with the stability of eigenvector/eigenvalue decomposition [25-27] on large datasets and due to the fact that the original Parafit code uses the algorithm from [28]. Therefore, the integration of the thoroughly tested LAPACK routines, apart from speed benefits, also yields increased numerical stability. We integrated AxPcoords and AxParafit into CopyCat [14]. Figure 1 provides a screen-shot of CopyCat whit a drop-down menu that allows the user to select AxParafit/AxPcoords for executing the analyses.

### Parallelization

AxPcoords requires less than 24 hours of run-time on a single CPU, even for distance matrices with several thousands of taxa. Therefore, we exclusively focused on the parallelization of AxParafit which requires run-times of several days or weeks on large datasets.

The execution time of Parafit depends on the sizes of input matrices *A*, *B*, and *C *with dimensions *n*_1_*n*_2_, *n*_4_*n*_1_, and *n*_3_*n*_2 _respectively (for details see [15]). The complexity is roughly *O*(*nonZero*(*A*)*n*_3_*n*_4_*n*_1_*p*). The term *n*_3_*n*_4_*n*_1 _is the complexity of the dense matrix multiplication in AxParafit. The variable *p *is the user-specified number of permutations that shall be executed (typically 99–9,999, not counting the original permutation) and *nonZero*(*A*) is the number of non-zero elements in the binary association matrix *A*. The program executes two main steps: the global test of co-speciation with complexity *O*(*n*_3_*n*_4_*n*_1_*p*) and the individual tests with complexity *O*(*nonZero*(*A*)*n*_3_*n*_4_*n*_1_*p*). Since in real-world analyses *nonZero*(*A*) ≫ 1 we only parallelized all individual tests of co-speciation which typically generate over 99% of the total computational load. Our approach represents a trade-off between the amount of programming effort required for the parallelization and the expected performance gains. Thus, initially the global test of co-speciation must be executed using the sequential version of AxParafit. The sequential program provides an option to conduct the global test, write a binary output file that can be used to start the parallel computation of individual host-parasite links, and then exit.

The statistical test of individual associations has been parallelized with MPI (Message Passing Interface) via a master-worker scheme. The parallelization is straight-forward since all tests of individual associations are independent from one another and can thus be computed independently on individual workers. Moreover, each individual test has approximately the same execution time, such that there are no problems due to load imbalance. The maximum number of CPUs that can be used by our parallelization is thus *nonZero*(*A*). However, this can be improved by using the ACML or MKL BLAS implementations that exploit fine-grained loop level parallelism on SMP (Symmetric Multi-Processing) architectures. This allows for a more efficient utilization of hybrid supercomputer architecture. Moreover, it might help to improve performance on huge datasets where SMP implementations can profit from super-linear speedups due to increased cache efficiency.

## Results and Discussion

The current Section is split into two parts: Part 1 describes the computational results while Part 2 outlines the substantial benefits of using AxParafit for large-scale empirical co-phylogenetic studies.

### Computational Performance

Here we provide performance data regarding the purely computational aspects of AxParafit.

#### Experimental Setup

To conduct computational experiments we used an unloaded system of 36 4-way AMD 2.4 GHz Opteron processors with 8 GB of main memory per node which are interconnected by an Infiniband switch. Parafit and DistPCoA were compiled using g77 -ffixed-line-length-0 -ff90-intrinsics-delete -03. AxParafit and AxPcoords were compiled with -03 -fomit-frame-pointer -funroll-loops and linked with the AMD ACML library. We also assessed additional compiler optimizations (-fomit-frame-pointer, -funroll-loops, -m64, -march = k8) with g77 for Fortran, which actually lead to performance decrease of Parafit and DistPCoA (data not shown).

In order to assess performance of AxParafit we extracted subsets from a large empirical dataset with more than 30,000 host-associate links (collected from entries in the EMBL database [29]), which we are currently analyzing with our tools. We sampled square association matrices *A*, i.e., *n*_1 _= *n*_2 _of dimensions 128, 256, 512, 1,024, and 2,048. The number *nonZero*(*A*) was 128, 256, 512, 1,024, and 2,048 respectively. The number of permutations *p *was set to 99, 99, 9, 2, and 2 respectively. A complete test on the dataset of size 4,096 was not conducted with Parafit due to the extremely long run-times on *n*_1 _= *n*_2 _= 2, 048 which already amounts to 19.9 days compared to 7.7 hours required by AxParafit.

To test AxPcoords we used the same compiler switches as indicated above and a subset of the square association matrices with *nonZero*(*A*) amounting to 512, 1,024, 2,048, and 4,096 respectively.

## Results

**Figure 2:**
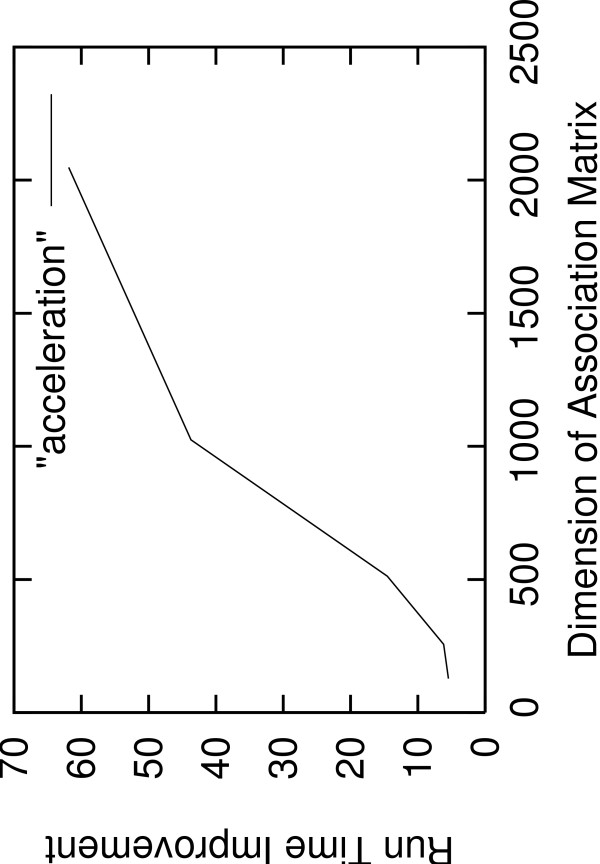
**Run Time Improvement Sequential AxParafit versus Parafit**. Run-time improvement of AxParafit versus Parafit for quadratic association matrices of dimensions 128, 256, 512, 1,024, and 2,048.

In Figure 2 we provide the sequential run-time improvement of AxParafit over Parafit. The acceleration obtained by AxParafit increases with growing dataset size and attains a factor of 61.86 on the association matrix of size 2,048. The increase of the performance improvement with growing dataset size is mainly due the larger efficiency of both our own optimizations as well as the cache blocking strategies used in the BLAS implementations.

**Figure 3:**
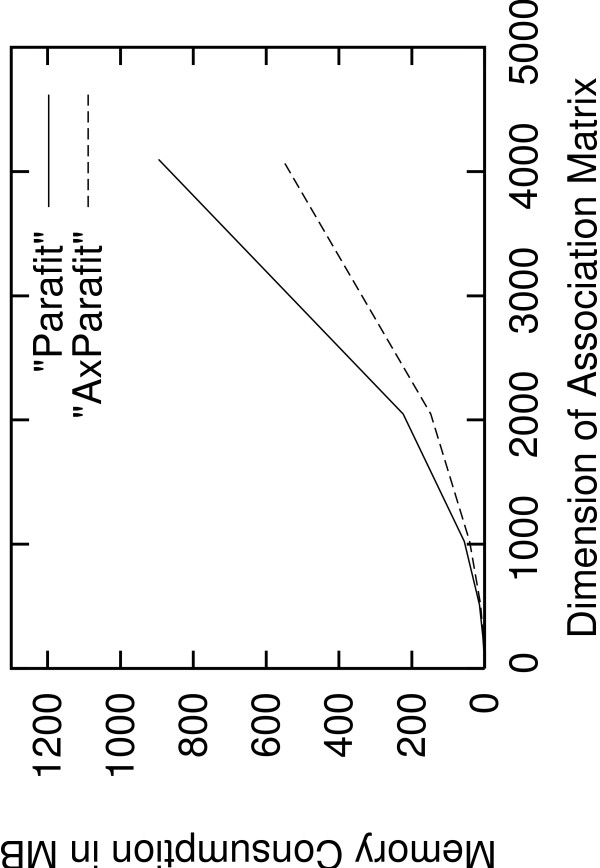
**Memory Consumption AxParafit versus Parafit**. Memory consumption of Parafit and AxParafit for quadratic association matrices of size 128, 256, 512, 1,024, 2,048, and 4,096.

**Figure 4:**
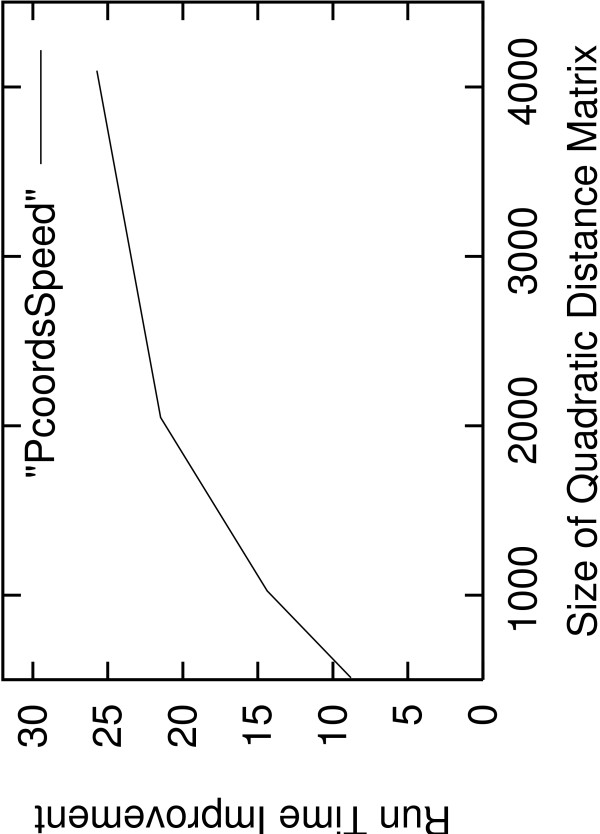
**Run Time Improvement Sequential AxPcoords versus DistPCoA**. Run-time improvement of AxPcoords versus DistPCoA for quadratic distance matrices of dimensions 512, 1,024, 2,048, and 4,096.

Figure 3 provides the memory use of AxParafit and Parafit in MB for quadratic *A*-matrices of sizes 128, 256, 512, 1,024, 2,048, and 4,096 (note that the dataset of size 4,096 was not run to completion). To test AxPcoords we used distance matrices of sizes 512, 1,024, 2,048, and 4,096. Run-time improvements range from 8.8 to 25.74. The run on 4,096 with DistPCoA apparently terminated but did not write a results file, most probably due to numerical instability (Pierre Legendre, personal communication). Figure 4 shows the run-time improvement of AxPcoords over DistPCoA for quadratic distance matrices of sizes 512, 1,024, 2,048, and 4,096. As already mentioned, the run on 4,096 with DistPCoA did not write a results file. Tests on smaller distance matrices e.g., of size 128 and 256 were omitted due to the low execution times which were below 10 seconds. On the largest matrix AxPcoords terminated within only 399 seconds as opposed to 10,268 seconds required by DistPCoA.

**Figure 5:**
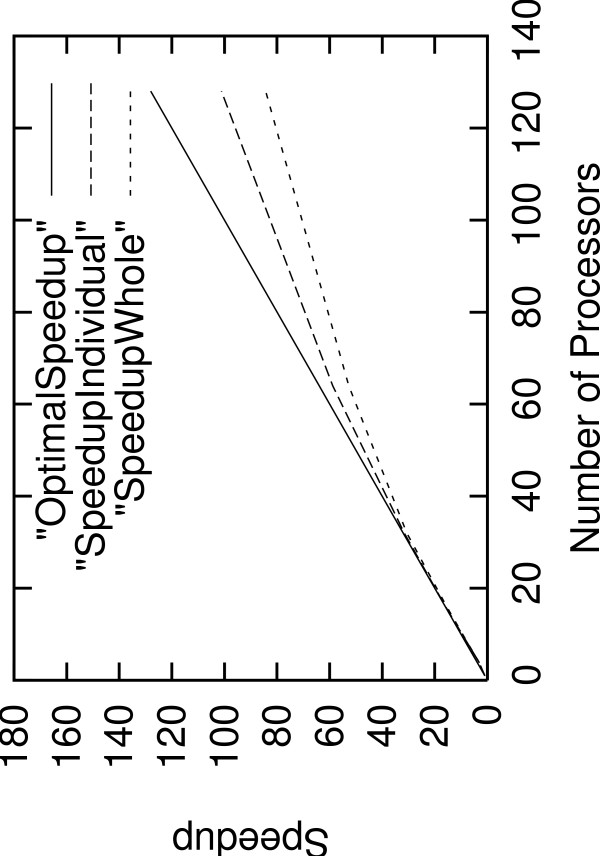
**Speedup of Parallel AxParafit**. Speedup of parallelized part and speedup for sequential plus parallel part of AxParParafit for a quadratic association matrix of size 512 on 4, 8, 16, 32, 64 and 128 CPUs.

We assessed scalability of parallel AxParafit using the association matrix *A *of size 512 on 4, 8, 16, 32, 64, and 128 processors with *p *= 99. Figure 5 provides the speedup with respect to the number of worker processes. We indicate speedup values for the parallel part (SpeedupIndividual, computation of individual host-parasite links) as well as for the sequential plus the parallel part of the program (SpeedupWhole), i.e., we added the sequential computation time for the global test to the parallel execution time. On 128 processors the computation took only 50 seconds. An analysis of this dataset with the sequential version of Parafit would take approximately 20 hours.

### A Real-World Example

In order to provide an example for the substantial benefits of performing a large-scale co-phylogenetic analysis with AxParafit we provide a real-world study on smut fungi and their host plants.

#### Experimental Data

We collected a large sample of associations of smut fungi and their host plants. Smut fungi comprise more than 1,500 species of obligate phytoparasites and are arranged in the taxa *Entorrhizomycetes*, *Microbotryales*, and *Ustilaginomycotina*. These parasites cause syndromes such as dark, powdery appearance of the mature spore masses or may even lead to plant deformation in some cases [30,31]. The *Ustilaginomycotina *also comprise obligate plant parasites with distinct morphology [30].

With a few exceptions, hosts of smut fungi belong to the Angiosperms [30]. For economically important hosts, such as barley and other cereals, smut fungi may cause considerable yield losses (see e.g., [32]). Phylogeny and taxonomy of genera and higher ranks has been derived from sound molecular and ultrastructural data in recent years (see [30] and references therein). However, apart from the work presented in [14], co-phylogenetic analysis of smut fungi have so far been restricted to single genera with comparatively few species [33,34].

In addition to the host plant index for European smut fungi [31,35] that has been used in [14], information on smut fungus-host plant associations was extracted from the following publications: Bauer et al. [36-38], Begerow et al. [33,39], De Beer et al. [40], Hendrichs et al. [41], Nannfeldt [42], Piepenbring [43], Scholz and Scholz [44], an unpublished manuscript by K. Vanky (Smut fungi of the Indian subcontinent; Vanky, personal communication), and Vanky and McKenzie [45]. Moreover, we included information contained in the "specific host" entries of the complete collection of core nucleotide sequences for *Entorrhizomycetes*, *Microbotryales*, and *Ustilaginomycotina *downloaded from GenBank [46] on September 01, 2007 (12,815 sequences). Parasite taxon names were corrected using Vanky's synonym-list [35]. Synonyms for host taxon names were obtained from Palese and Moser [47].

Including synonyms, our data set contained 3,912 different fungus-plant associations. In order to retrieve taxon IDs and to construct taxonomy trees for hosts and parasites [14], we used the NCBI taxonomy release of September 01, 2007. For host and parasite species names that were not found in the NCBI taxonomy, the search was repeated after reducing the taxon name to the respective genus. In this way, a total of 2,362 different associations could be identified that covers 413 smut fungi and 1,400 host plants. Thus, the dataset assembled was more than *three times *larger than the one recently analyzed in [14], which contained 645 associations, corresponding to 140 smut fungi and 437 host plants. The Parafit analysis of this comparatively small dataset took already more than a week. For both hosts and parasites, two trees were constructed, one tree with branch lengths corresponding to the "true" (denoted as W for Weighted) taxonomical distance [14] and one with all branch lengths set to 1 (denoted as U for Un-weighted/Uniform). As outlined on page 4 the computational complexity of AxParafit is *O*(*nonZero*(*A*)*n*_3_*n*_4_*n*_1_*p*) and thus the execution time requirements for this larger dataset increase significantly.

#### Inference with AxParafit

Production runs with Parafit and AxParafit on an initial version of our dataset were started on August 29, 2007. While the Parafit inferences with 99 permutations on this initial dataset were still running at the time of writing this manuscript(September 9, 2007), the parallel AxParafit run with 99 permutations terminated within less than 480 seconds on 128 CPUs of the Infiniband cluster. This made the results available immediately and allowed us to identify a bug in the data collection script. The buggy version of this script did not take the presence of non-unique scientific taxon names, (e.g.,*Setaria *(*Magnoliophyta, Poales*) and *Setaria *(*Nematoda, Filarioidea*)) into account to identify NCBI taxon IDs. Such errors are unfortunately typical and frequent in Bioinformatics analysis pipelines. As a typical example of such errors consider the retraction of "Measures of Clade Confidence Do Not Correlate with Accuracy of Phylogenetic Trees" by Barry G. Hall due to an error in a perl script [48].

In addition to the rapid detection of input data errors, the significant performance gains obtained by sequential optimization and parallelism allow for the assessment of different program parameters and analysis options, such as trees with different patristic distances (U and W trees) as well as the impact of the number of permutations on the results (AxParafit was run with 99/999/9,999 permutations on the U and W data), i.e., a significantly more thorough and detailed analysis.

The absolute execution times for AxParafit on 128 CPUs for 99/999/9,999 permutations are indicated in Table 1. Essentially, 99 permutations could be conducted within 7 minutes, 999 permutations in much less than 2 hours, and 9,999 permutations overnight in about 12 hours such that the whole study, including the detection of the script error and the analysis of the results could be completed in less than a week. As indicated in Table 2 there are a number of links (max. 48 out of 2,362 ≈ 2%) that are not uniformly significant or uniformly insignificant at low *p*-values between analyses with a distinct number of permutations. AxParafit therefore allows for rapid and much more thorough computation and analyses of large co-phylogenetic datasets. The results indicate that U-based analyses are in general more sensitive to the number of permutations than W-based runs. Note that the number of host/parasite eigenvectors for U (1,390/411) was higher than for W (1,200/372), which explains the longer execution times and potentially the larger differences in significance values.

**Table 1:** Empirical Data Study: Parallel AxParafit Execution Times

# Permutations	99	999	9,999
W	355 secs	3,759 secs	39,170 secs
U	451 secs	4,441 secs	47,221 secs

Parallel execution times in seconds for AxParafit on 128 CPUs for 99/999/9,999 permutations.

**Table 2:** Empirical Data Study: Impact of the Number of Permutations

# Permutations	99/999/9,999		99/999		99/9,999		999/9,999	
Tree	W	U	W	U	W	U	W	U

p = 0.01	16	48	14	35	13	36	5	25
p = 0.02	7	27	5	27	6	27	3	0
p = 0.03	4	22	3	17	4	19	1	8
p = 0.04	2	18	1	17	2	18	1	1
p = 0.05	1	8	0	8	1	7	1	1

The table outlines the impact of the number of permutations on the distribution of significant and insignificant links for distinct *p*-values. Column (99/999/9,999) indicates the number of links that have a different significance than at least one of the other runs.

Table 3 indicates the number of different significant links between the U- and W-based analyses for various *p*-values. The table indicates that there is no clear tendency for differences to decrease with increasing number of permutations.

**Table 3:** Empirical Data Study: Differences between U and W-based Analyses

p-value	0.01	0.02	0.03	0.04	0.05
99	91	60	42	29	16
999	76	54	44	15	10
9,999	84	51	51	13	8

The table shows the differences between U and W-based analyses of individual associations for different *p*-values and numbers of permutations.

#### Biological Interpretation of Results

**Figure 6:**
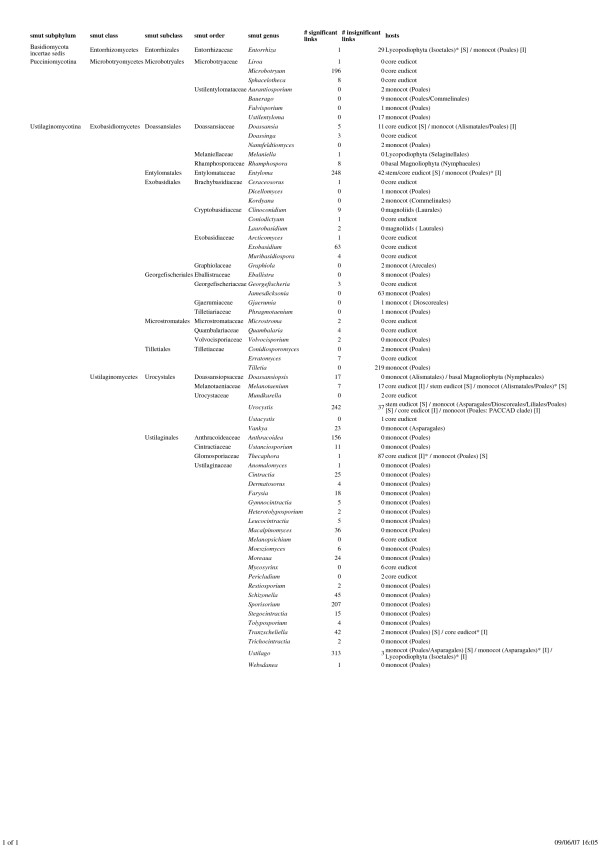
**Overview of the Results for Individual Host-Parasite Links based on the Smut Fungi Genera**. Major taxonomic groups of host and parasites are indicated according to the NCBI taxonomy release used. Significant and insignificant associations are indicated as [S] or [I] respectively. Stars denote to doubtful associations.

In the following, we focus on the results obtained with 9,999 permutations and branch lengths scaled in terms of taxonomical distances (W-labeled results). The global test indicates a highly significant co-phylogenetic relationship (*p *= 0.0001). An overview of the results for individual host-parasite links based on the smut fungi genera is provided in Figure 6. Major taxonomic groups of host and parasites are indicated according to the NCBI taxonomy release used. Based on a significance threshold of *p *= 0.05 and the ParafitLink1 statistics [15], a total of 578 insignificant and 1,784 significant associations is obtained. As in our earlier study [14], genera of smut fungi are rather uniform with respect to their significance values, which facilitates the identification of a general distribution pattern with respect to significant and insignificant links, i.e., the "deep co-phylogeny" of smut fungi.

The single most important factor appears to be whether the hosts belong to the monocots (i.e.,*Liliopsida*) or not. *Entorrhiza *species, which are taxonomically isolated, mostly are linked with monocots (*Poales*) and do thus not contribute significantly to the overall fit between host and parasite phylogenies. In the case of *Microbotryales*, the majority of taxa are pathogenic of core eudicots, resulting in significant links. Fewer associations with monocots (mostly *Poales*) are present, which are considered insignificant. The same pattern can be observed in the class *Exobasidiomycetes *within *Ustilaginomycotina*: A minority of host-parasite links is within monocots (*Poales*, but also other orders), which are considered insignificant, whereas the associations with other hosts (*Selaginellales*, basal *Magnoliophyta*, magnoliids, and stem and core eudicots) are significant. Inverse relationships are present in the class *Ustilaginomycetes *within *Ustilaginomycotina*. Here, most species infect monocots, mainly *Poales*, significantly increasing the congruence between host and parasite taxonomy trees, whereas the associations with core eudicots appear to be insignificant.

Accordingly, the current analysis that is based on a considerably larger empirical sample (e.g., 66 instead of 25 included genera of smut fungi) confirms earlier results [14]. Therefore, we can generalize the observation that the difference between *Poales *and non-*Poales *hosts is crucial for the distribution of significance values to the distinction between monocot and non-monocot hosts. We also observe a small number of exceptions from this general pattern. For instance, in *Urocystis *(*Ustilaginomycetes*), which occurs on a variety of host groups, the links with stem eudicots (species of *Ranunculaceae*) are significant, and a single link with monocots (PACCAD clade within *Poaceae*) is judged as insignificant. Thus, rather subtle details of the host-parasite relationships, such as the presence of *Urocystis *on several closely related Ranunculaceae hosts and its presence on distantly related hosts within *Poaceae*, are recognized by the AxParafit algorithm, and the uniform overall pattern does not merely reflect the relatively low topological resolution present in the taxonomy trees.

Some of the results obtained may also be due to flaws in the taxonomy of the species included, particularly in the nomenclature of the parasites. For instance, *Entorrhiza isoetis *is most likely conspecific with *Ustilago isoetis *[31]. At present it is even doubtful whether this species belongs to smut fungi (R. Bauer, personal communication). Thus, the associations with Isoetes (*Lycopodiophyta*) mentioned in Scholz and Scholz [44], which show different significance values than the majority of hosts links in either *Ustilago *or *Entorrhiza*, are dubious. Likewise, the exceptional associations of *Entyloma *with monocots are probably due to species names that would need to be recombined into genera of the *Georgefischeriales *[37]. Whereas these flaws have to be corrected by considering more comprehensive lists of species and synonyms in monographs and in future releases of the NCBI database, it is apparent that neither the highly significant overall co-phylogenetic relationship nor the general pattern regarding individual host-smut fungus links would be affected by the removal of the doubtful associations. Rather, their influence is overcome by the large total sample size; for each parasite genus dubious links are few relative to the total number of links or not present at all. Likewise, there are few differences in the significance between analyses with a distinct number of permutations (see Table 2). Discrepancies between U and W are also comparatively small (see Table 3). With 9,999 permutations, they are restricted to four genera of smut fungi and only affect hosts, such as *Urocystis *on monocots in *Asparagales *and *Dioscoreales *(details not shown), with an intermediate taxonomic position.

The analysis process presented here underlines the advantage of the large-scale approach to co-phylogenetic tests, that is enabled by AxPcoords/AxParafit. Furthermore, because many problems are more easily recognized after conducting preliminary runs, re-analysis after applying corrective measures may be necessary for many empirical datasets. Thus, efficient implementations and parallelism are of great practical importance for the analysis pipeline.

## Conclusion

We have produced highly optimized and efficient implementations of the two most compute-intensive components for P. Legendre's statistical test of host-parasite co-speciation. The parallel implementation of AxParafit scales well up to 128 CPUs on a medium-size dataset. AxParafit and AxPcoords have been integrated into the CopyCat tool and are freely available for download as open source code.

Future work will mainly cover large-scale production runs with AxParafit.

## Availability and Requirements

The source code and some of the test datasets are available at ic .

The datasets and results of the empirical study on smut fungi are also available at this site. It also provides several pre-compiled binaries for Windows, MAC, and Linux/Unix platforms.

AxParafit can be compiled as stand-alone application without making use of either ATLAS, MKL or ACML. AxPcoords requires either MKL, ACML, or the GNU scientific library.

The new CopyCat version that uses AxParafit and AxPcoords is available at .

## Authors' contributions

AS ported the programs from Fortran to C, optimized the C code, integrated the BLAS and LAPACK packages, parallelized the program and performed the computational experiments. AFA and JMK carried out the integration into CopyCat. AFA, JMK, and MG assembled the Binaries for various platforms and provided scripts to conduct the computational experiments. MG assembled the test datasets. AS and MG conducted the empirical study on smut fungi and their hosts. AS, AFA, JMK, and MG wrote the manuscript. All authors read and approved the final manuscript
